# Characterization of tonsillar IL10 secreting B cells and their role in the pathophysiology of tonsillar hypertrophy

**DOI:** 10.1038/s41598-017-09689-x

**Published:** 2017-09-11

**Authors:** Lindybeth Sarmiento Varon, Javier De Rosa, Andrés Machicote, Luis Ariel Billordo, Plácida Baz, Pablo Mariano Fernández, Isabel Kaimen Maciel, Andrés Blanco, Eloísa I. Arana

**Affiliations:** 1 Institute of Immunology, Genetics and Metabolism (INIGEM), Clinical Hospital, University of Buenos Aires, National Council for Scientific and Technological Research, Buenos Aires, Argentina; 2 Department of Immunology, School of Medicine, University of Buenos Aires, Buenos Aires, Argentina; 3 Otolaryngology Service, Clinical Hospital, University of Buenos Aires, Buenos Aires, Argentina; 4 Institute of Otolaringology Arauz, Buenos Aires, Argentina

## Abstract

The comprehension of unconventional immune functions of tonsillar B cells, their role in tolerance induction and protective immune responses, is crucial to unveil the dynamic interactions of the upper aero digestive tract with polymicrobial commensal flora and pathogens, in health and disease. Here, we describe the kinetics of IL10 intracellular expression and compare it with that of cytokines known to be produced by tonsillar B cells. Additionally, we detected a relevant proportion of IL17-expressing tonsillar B cells, which has not previously been reported. We immunophenotyped tonsillar IL10-expressing B cells (B10) and observed IL10 production in activated B cells at every developmental stage. Finally, we identified a relationship between decreased B10 percentages, increased proportion of the germinal centre (GC) population and hypertrophied tonsils (HT). Our findings provide greater insight into the role of B10 in GC reactions and characterized their involvement in the pathogenesis of tonsillar dysfunction.

## Introduction

Tonsils are the first sites where microbial and environmental antigens (Ags) entering through the upper aero digestive tract, are managed in the human body. Suitably located at the entrance of the pharynx to grant immunologic protection, tonsils are continually exposed to dietary and airborne Ags. While lymph nodes depend on antigenic delivery through afferent lymphatics, tonsils have Ag-retaining crypts that increase their surface and enable the direct transport of the Ags deposited there, a feature that favours Ag presentation to lymphoid cells on the interior. These lymphoid cells are predominantly B cells (55% ± 15% of all tonsillar lymphocytes, depending on age). B cell receptor (BCR) engagement by Ags followed by cognate T follicular cell help (Tfh) drives the proliferation of Ag-specific naive B cells in germinal centres (GCs). GCs are microenvironments within secondary lymphoid tissues (*i.e*., tonsils) that are critical for memory B cell and plasma cell generation^1^. Therefore, the tonsils are sites of increased expansion of B cell diversity and memory^2^. However, tonsillectomy is indicated for patients with a history of recurrent or chronic tonsillitis (RT) or for those with obstructive symptoms from tonsillar hypertrophy (HT, no history of recurrent infections in the case of the samples in the present, unless indicated otherwise)^3^. The pathogenesis of these conditions remains largely unknown. Thus, human tonsils are unique inductive immune sites for B cell responses, provided that these organs operate properly.

Such responses are not restricted to antibody production and Ag presentation. B cell-derived cytokines are crucial for multiple aspects of immunity^4^, and new B cell subpopulations have consequently been identified. For example, a subset of B cells that produce the anti-inflammatory cytokine IL10, designated Bregs by Mizoguchi^5^, has been reported in mice and humans. In humans, multiple studies have shown that these cells are either numerically or functionally deficient in several diseases^6–11^ and are also related to renal transplant tolerance^12^. More recently, B cells have also been demonstrated as a source of the pro-inflammatory cytokine IL17 -which was initially thought to be exclusively secreted by Th17 cells- in a murine model^13^ and in human PBMCs^14^.

Tonsillar immune actions (anti- and pro-inflammatory) must be tightly regulated to balance the protection against virulent germs and the tolerance to harmless flora and innocuous Ags in the oropharynx. We proposed that because tonsils are B cell organs, the B10 population plays a role in such regulation. Despite the relevance of the issue in light of the importance gained by the mucosal route of vaccine administration, tonsillar B10 has not yet been fully characterized.

In the present study, we determined the phenotype of tonsillar B10, analysed the kinetics of IL10 intracellular expression and compared these results with those of other cytokines produced by tonsillar B cells. Notably, this study is the first to report a relevant proportion of IL17-expressing tonsillar B cells. Finally, we discuss the physiological relevance of B10 *in vivo*, as samples donated from HT patients presented a lower proportion of B10 and a higher fraction of GC than samples donated from RT patients.

## Results and Discussion

### Characterization of IL10 production by B cells from human tonsils

**Figure 1:**
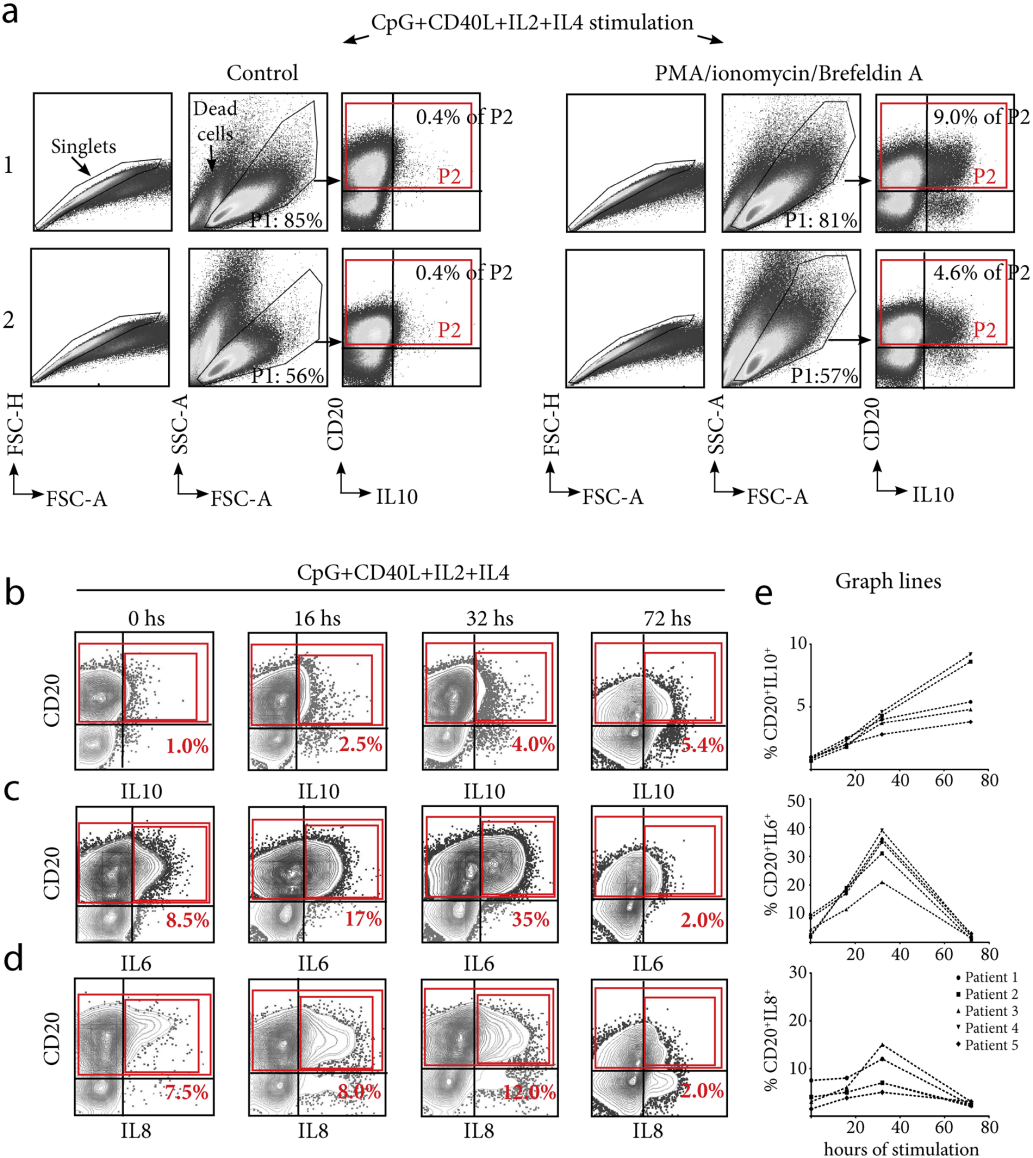
Cytokine intracellular expression by tonsillar B cells. (**a**) B cell cytoplasmic IL10 expression by 2 tonsil donors. Tonsil 1 was from a patient with RT (representative of this group) and 2, representative from patients with HT. TMC were cultured for 72 hs on CpG+CD40L+IL2+IL4. The cells were subsequently stained for surface CD20 and intracellular IL10. To detect IL10, the samples shown in the right panels were stimulated with PMA/ionomycin/Brefeldin A for the last 5 hs. Control sets (left panels) did not receive such treatment. P1 denotes the percentage of living cells determined using FSC-SSC dot plot profiles within the singlets gate. P2 denotes the percentage of B10 among CD20^+^ B cells. Total CD20^+^ B cells are indicated within the large red gate representing 100% of B cells. The data from one experiment representative of 12 independent experiments performed with different individuals are presented. (**b**), (**c**) and (**d**) Kinetics of cytoplasmic IL10, IL6 and IL8 expression by tonsillar B cells, respectively. The cells were stained for surface CD20 and intracellular IL10, IL6 or IL8. The percentages designate cytokine-producing B cell frequencies among CD20^+^ B cells. Total CD20^+^ B cells are indicated within the large red gate representing 100% of B cells. CD20^+^ IL10^+^ (**b**) CD20^+^ IL6^+^ (**c**) and CD20^+^ IL8^+^ (**d**) detected at the different time points of culture, indicated in the top panel, are specified with the small red gate. TMC were cultured and stimulated as in (**a)**. The data from one experiment representative of 5 independent experiments performed with different individuals are presented. (**e**) Line graphs plotting the results of 5 different individuals for IL10, IL6, and IL8 kinetics, respectively.

Co-treatment with CpG and CD40L is a potent method of inducing IL10 production by circulating B cells, peaking at 72 hours hs post stimulation^15^. As tonsillar B cells express high levels of TLR9^16^, we used this approach to examine their production of IL10 at the single cell level using FACS. IL2 and IL4 were also added to the culture, as they promote the survival of all B cell subsets^17^. The cells that were already dead prior to permeabilization were excluded from the analysis based on their forward- and side-light scatter properties (P1, Fig. 1a), as we confirmed that the fixation procedure utilized appropriately preserved the cell morphology. Such scatter-based assay for distinguishing the viability of untransformed human B cells showed consistency with the results obtained from FITC-annexin staining^18, 19^. We previously confirmed the results using dead cell staining and showed that dead cell discrimination by forward/side scatter presented excellent consistency with the results obtained using 7 amino-actinomycin D (7AAD) staining specifically for tonsillar B cells^17^. We observed a substantial production of IL10 (Fig. 1a). Tonsillar and circulating B cell subsets exhibit differences with respect to the kinetics of activation^17^; therefore, we replicated the assay at earlier time points. We observed a trend similar to that reported for circulating B cells^6, 7, 20^. While the proportion of tonsillar B10 steadily increased between 16 and 72 hs in all cases (Fig. 1b and e), the proportion of B10 detected at the different time points varied between donors and the cause of each surgery, an issue that will be addressed in the following sections. As a control for the appropriate stimulation of tonsillar B cells, we also measured IL6 and IL8 expression, as these are cytokines with faster release kinetics than IL10^16^. We confirmed that tonsillar B cell cultures readily produced other cytokines at early time points at which IL10 was barely detected (Fig. 1c, d and e). We concluded that comparable signals induce circulating B10 and tonsillar B10 to express IL10 *in vitro* in a similar fashion in terms of kinetics and overall percentage.

Notably, unlike IL10, in some cases, significant levels of IL6 and IL8 could be detected at the single cell level without CpG/CD40L stimulation (at 0 hs, ~8.5% and ~7.5%, respectively, for the patient shown in Fig. 1c and d), confirming our previous reports in relation to the capacity of tonsillar B cells to promptly produce good amounts of such cytokines on a per cell basis, reflecting their adaptation to a strong and immediate inflammatory response on site^17^. At 16 hs post stimulation, ~17% of the tonsillar B cell population had become IL6-secreting cells, ~8% had become IL8-secreting cells, and ~2.5% represented the B10 population. At the following time point, 32 hs post stimulation, more than one-third of B cells in culture produced IL6, whereas ~12% of B cells exhibited intracellular IL8, and IL10 could be detected in ~4% of B cells. Finally, at 72 hs post stimulation, approximately ~2% of the B cells in culture expressed either IL6 or IL8. Thus, in terms of the relative kinetics of production, these findings were consistent with those of Gantner F. *et al*.^15^ using an alternative method to measure cytokines and further confirmed the ability of B cells to secrete pro and anti-inflammatory cytokines, as recently reviewed by Shen and Fillatreau^4^. With regard to the percentage of IL6- and IL8-secreting cells at different time points, the results varied across individuals; therefore, we showed the contour plots of a single patient and graph lines plotting the results of 5 different patients for the cytokine kinetics. Further work into recruiting more patients is required to perform meaningful statistics and accurately elucidate putative associations between the proportions and levels of pro-inflammatory cytokine expression and causes of surgery, as described below for IL10.

**Figure 2:**
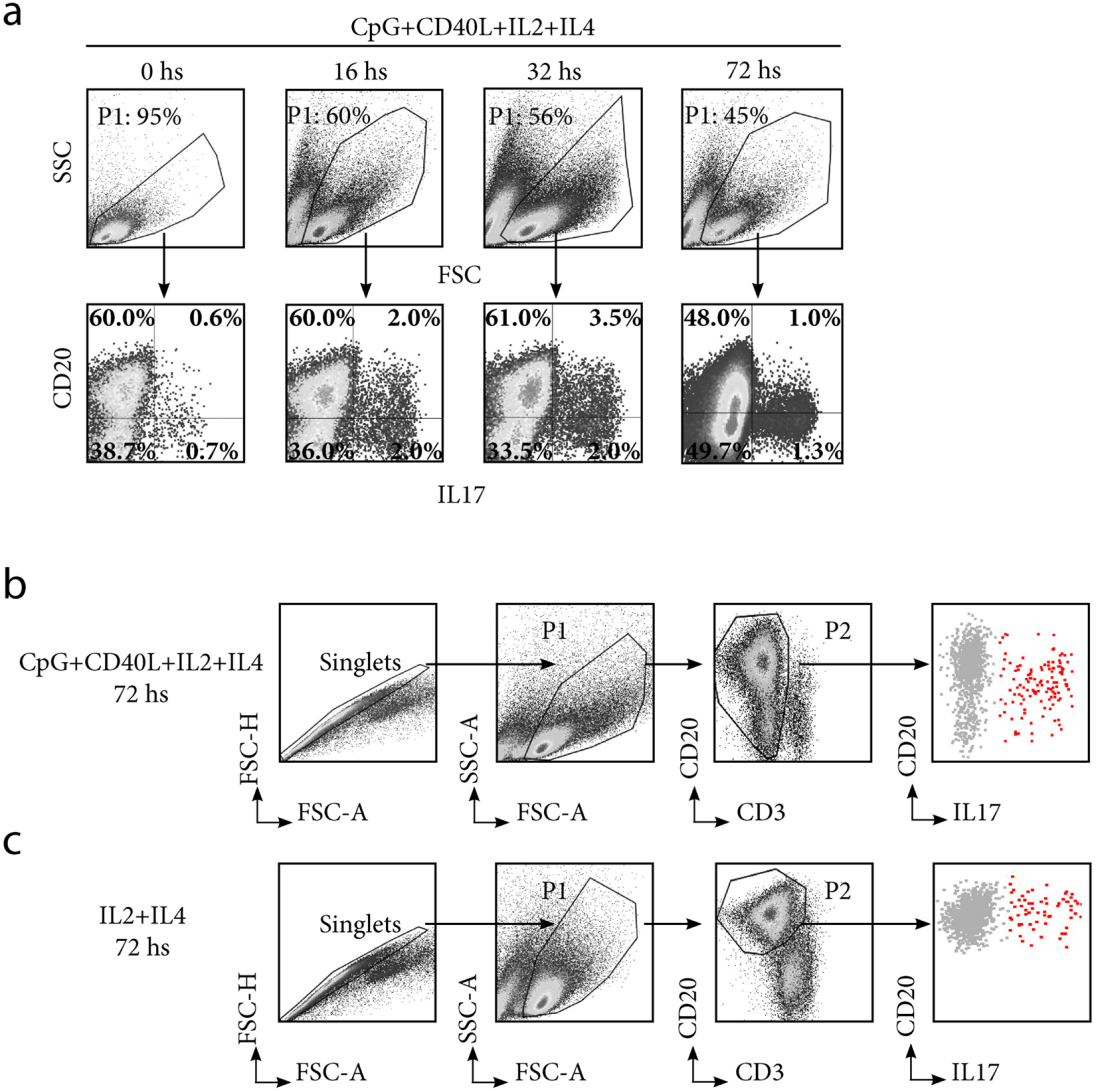
Cytoplasmic IL17 expression by tonsillar B cells. (**a**) Kinetics of cytoplasmic IL17 expression. TMC were cultured for the time points indicated at the top of each panel, on CpG+CD40L+IL2+IL4. To detect IL17, the cells were stimulated with PMA/ionomycin/Brefeldin A for the last 5 hs and subsequently stained for surface CD20 and intracellular IL17. Upper panels: P1 denotes percentage of living cells determined using FSC-SSC dot plot profiles within the singlets gate (determination of singlets gate not shown). Lower panels: dot plots showing IL17 and CD20 expression from TMC gated on the P1 population. Percentages designate frequencies of the populations in each quadrant. Data from one experiment representative of 5 independent experiments performed with different individuals are presented. (**b**) TMC were cultured for 72 hs on CpG+CD40L+IL2+IL4 and PMA/ionomycin/Brefeldin A for the last 5 hs. The cells were subsequently stained for surface CD20 using a different fluorophore than used in (**a**), CD3 and intracellular IL17. P1 indicates living cells determined using FSC-SSC dot plot profiles within the singlets gate and P2 indicates the CD3^-^ CD20^+/down^ cells. CD20^+/down^L17^+^ cells are shown in red. (**c**) TMC were cultured for 72 hs on IL2+IL4 and PMA/ionomycin/Brefeldin A for 5 hs. The cells were subsequently stained for surface CD20, CD3 and intracellular IL17. P1 indicates living cells determined using FSC-SSC dot plot profiles within the singlets gate and P2 indicates CD20^+^ cells. CD20^+^IL17^+^ cells are shown in red.

Importantly, we also observed that tonsillar B cells produced considerable amounts of IL17 (Fig. 2). IL17 secretion by T cells (Th17) has been implicated in defence against extracellular pathogens and the pathogenesis of inflammatory diseases. As tonsillectomy generally indicates the chronic inflammation of tonsils due to RT and/or HT, our observation seems logical. While Th17 cells have been the subject of multiple studies, reports on the secretion of IL17 by B cells are scarce^13, 14^. Hence, these results complement the reports of previous studies, extending such capacity to B cell rankings in the human mucosal immunology system. When we analysed the kinetics, IL17 secretion from tonsillar B cells peaked upon 32 hs of CpG and CD40L stimulation.

It was not feasible to detect whether B10 co-expressed any of these pro inflammatory cytokines, as intracellular cytokine levels at the 72 hs time point were quite low for the whole culture. Moreover, as evidenced in Figs 1 and 2a, this late time point was particularly affected by the down-modulation of surface CD20 in response to different stimuli, and this effect has been extensively described previously^21, 22^. As the present study is the first report on tonsillar IL17-expressing B cells and their progress in time, we seek to improve such determination at 72 hs by excluding CD3^+^ cells as likely contaminants when CD20 expression is lost (Fig. 2b). Finally, we supplemented cultures with just IL2 and IL4, which prolong cell survival with minimal stimulation, thus circumventing CD20 downregulation and confirming IL17 expression by tonsillar B cells under those conditions (Fig. 2c).

Collectively, these results demonstrated that despite disease-associated changes, such as RT and HT, B cells from those damaged tonsils preserve their immune competence to secrete a number of cytokines. Considering that B cells comprise the majority of tonsillar lymphocytes^17^, an understanding of the relative levels of the different cytokine expressing B cell subsets and their association with tonsillar pathology and oral microbiome certainly requires further investigation. Importantly, a comprehensive picture of the effect of B cell cytokines on nearby cells and their microenvironment may inform new therapies for paediatric patients.

### Phenotype of IL-10-competent tonsillar B cells

**Figure 3:**
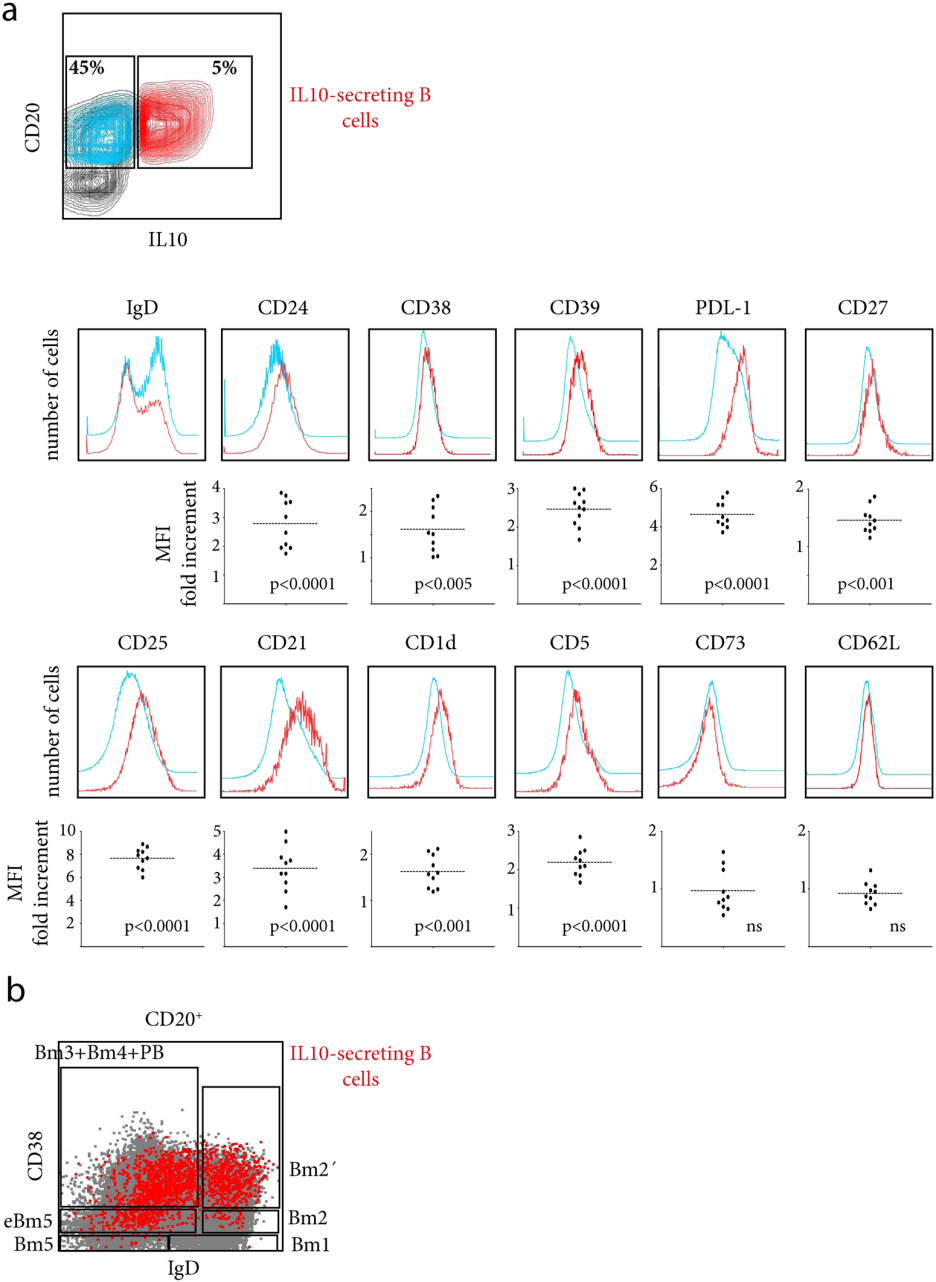
Presence of IL10 secreting B cells at every developmental stage after activation. (**a**) Upper panel, representative flow cytometry contour plots for CD20^+^ IL10^+^ (red) vs. CD20^+^ IL10^-^ (light blue) cells. Lower panels, extended phenotyping of IL10^+^ (red histogram) vs. IL10 ^-^ B cells (light blue histogram). TMC were cultured in the presence of CpG+CD40L+IL2+IL4 for 72 hs and subsequently stimulated with PMA/ionomycin/Brefeldin A for the last 5 hs. The data from one experiment representative of 10 independent experiments performed with different individuals are presented. A graph showing the MFI fold increment (median of fluorescence of B10/median of fluorescence of IL10^–^ B cells) for each independent experiment for the respective molecule is shown below each representative overlay of histograms. p results correspond to a one-sample t-test, reflecting a null hypothesis that there were no differences in MFI between B10 and IL10^–^ B cells (expected ratio of 1 for the test). (**b**) Dot plot depicting B10 cells (in red) within the classification of human tonsillar B cell subsets based on the expression of IgD/CD38 as in^20^ the graph obtained using the backgating option. TMC were cultured in the presence of CpG+CD40L+IL2+IL4 for 16 hs and subsequently stimulated with PMA/ionomycin/Brefeldin A for the last 5 hs. The data from a patient with a significant level of IL10 expression at 16 hs are shown as big dots to highlight the scattered B10 cell pattern on the CD20^+^ population, which appears as a solid grey cloud reflecting the amount and size of the dots. (**a**) and (**b**) All results correspond to events within singlets and viable gates.

The better understood and most studied regulatory mechanism of B cells is mediated by IL10; therefore circulating human B10 had been exhaustively phenotyped^6, 7, 19^. Considering the critical role that tissue environment imprints on the different immune cellular subsets, we characterized tonsillar B10. We performed a multiparametric FACS assay on tonsillar B cells to analyse the expression of numerous cell surface markers previously associated with an IL10-secreting profile in B cells isolated from PBMCs. Compared with their IL10^–^ B cell counterparts, tonsillar B10 expressed statistically significant higher levels of CD24, CD27, CD38, CD39, PDL1, CD25, CD1d, CD5, and CD21, consistent with the observations previously published for circulating B10 under similar conditions (Fig. 3a) and extensively reviewed in ref. 23. However, B10 did not significantly differ from IL10^–^ B cells in terms of the expression of CD73, CD62L and IgD (Fig. 3a), the latter presenting bimodal expression for both populations in all cases.

The Bm1-5 classification system^24^ is generally used to identify the multiple B cell subsets present in tonsils. In terms of development, every stage of B cell differentiation is represented in secondary lymphoid tissues, such as tonsils, and these stages can be cytometrically identified as Bm1: IgD^+^CD38^−^ (virgin naïve cells); Bm2: IgD^+^CD38^+^ (activated naïve cells not distinguishable from unswitched memory B cells with this classification); Bm2′: IgD^+^CD38^++^ (pre-GC cells indistinguishable from transitional B cells); Bm3 and Bm4: IgD^−^CD38^++^(same phenotype for GC cells and plasmablasts); and eBM5 and Bm5: IgD^−^CD38^+/−^ (memory cells). To further evaluate where the B10 subset would fall under such classification scheme, we stimulated the cells for a shorter period of time (16 hs), as such brief stimulation is adequate for B10 to produce IL10 without inducing changes in the overall phenotype of the CD20^+^ culture. We detected B10 within the Bm2, Bm2′, Bm3, Bm4 eBm5 and Bm5 subsets (Fig. 3b). The present study is not the first to report that B10 are diffusely scattered throughout the B cell lineage. According to previous reports, B10 could be present within the IgM memory^10^, GC-derived plasmablast^11^, and immature transitional^6, 10^ compartments. In fact, whether the immature transitional compartment effectively contains B10 remains controversial^25^. As previously indicated by Iwata and colleagues^7^, we confirmed (data not shown) that B10 do not express CD10 at the high levels required for classification as immature precursors. Through the CD10 staining, we could distinguish between pre-GC cells and transitional B cells. Therefore, we can ascertain that tonsillar B10 are pre-GC cells, indicating prior Ag stimulation. In any case, this plethora of phenotypes can only be plausible if B10 cells experience the entire differentiation programme once activated, secreting IL10 at every developmental stage, suggesting that these cells are pre-destined for this function, i.e., these cells comprise a separate lineage.

### Hypertrophied tonsils with intensively active germinal centres are characterized by a reduced B10 population compared with tonsils excised as a result of recurrent tonsillitis

**Figure 4:**
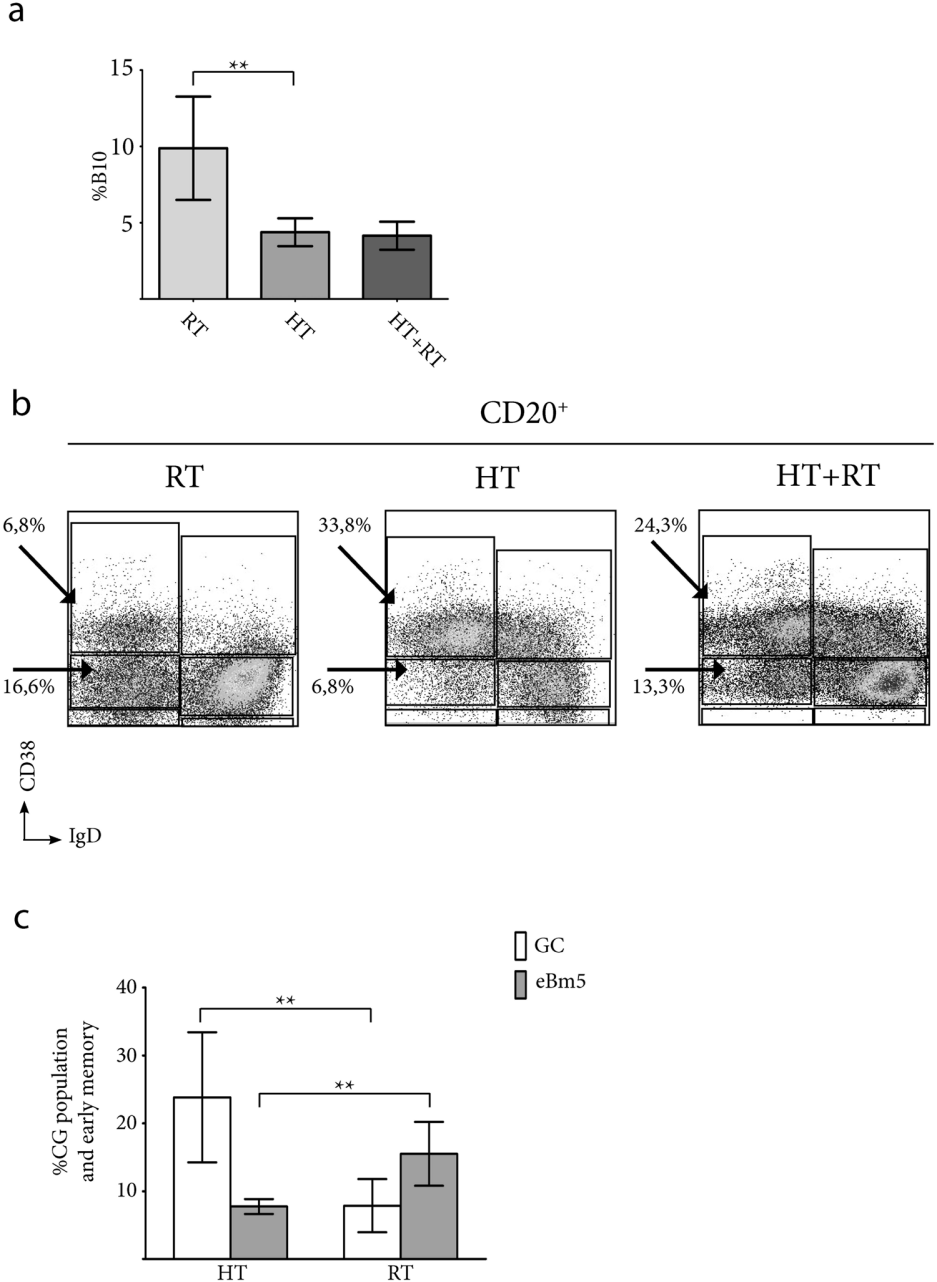
Physiological relevance of IL10-secreting B cells in tonsils. (**a**) The data show the mean %B10 ± S.D pooled from the independent experiments performed with tonsils from 12 different individuals grouped according the cause of surgery (RT, n = 5; HT, n = 5; HT+RT, n = 2; see Table 1). The proportion of B10 was determined using FACS, and TMC were stained for surface CD20, CD3 and intracellular IL10 after culture for 72 hs in the presence of CpG+CD40L+IL2+IL4, followed by stimulationwith PMA/ionomycin/Brefeldin A for the last 5 hs. (**b**) Dot plot depicting the percentage of the tonsillar B cell subsets for the 3 groups of patients (a selected individual for RT; HT; HT+RT). Fresh TMC were stained for CD20, IgD, and CD38 for the patients detailed in 3a. Arrows and percentages indicate frequencies of GC and early memory B cell populations. (**c**) The data show the mean percentage of the frequencies of GC and early memory ± S.D pooled from independent experiments performed with tonsils from the same 12 individuals detailed in 3a. Statistical analysis of the results in 3a and 3c was performed using unpaired Student’s t test**p < 0.005.

As previously mentioned, the samples donated for the present study were derived from several diseases: RT, HT (no history of recurrent infections), and eventually, HT combined with RT (Table 1). The differences in the pathologies that led to surgery were useful to assess the physiological relevance of tonsillar B10. We only received 2 samples from patients with HT combined with RT; thus, a relevant statistical analysis with that group was not feasible, and we showed the results from these patients but limited the discussion to the groups that included a majority of patients, granting statistical relevance to the observations. For the patients listed in Table 1, we scored the percentages of B10 following the gating strategy depicted in Supplementary Fig. S1. As we used cell shape to distinguish dead/live cells as opposed to using a more reliable fixable viability dye, we cannot rule out contaminating dead cells in the P1 gate, which could non-specifically bind anti-IL10. The isotype control (see Supplementary Fig. S1) evidenced that such fraction accounted for less than 0.5% of the cells. Interestingly, HT tonsils accounted for samples with a significantly lower percentage of B10 cells compared to those from RT (Fig. 4a, p < 0.005) and presented a significantly higher proportion of the GC population (Bm3 and Bm4, Fig. 4b; p < 0.005 Fig. 4c) than those from children with RT. Such comparative increment of the GC percentage was not accompanied by a proportional growth of the memory B cell population in those HT samples. In contrast, these samples displayed a significantly lower percentage of the eBm5 subset than RT samples (Fig. 4b; p < 0.005 Fig. 4c), indicative of a putative blockade between the GC and memory B cell stages on HT patients. This finding is consistent with the results of a previous report^26^, in which the authors compared histological cross sections of the surgical samples from HT and RT patients. Notably, they found that the number of GCs is the only histopathological criterion that can be used to differentiate the samples from the two groups. In this context, Achour*et al*.^27^ recently demonstrated a role for tonsillar B10 in controlling the functions of Tfh cells. The results of the present study extended these findings, uncovering a correlation *in vivo*, as lower the proportion of B10 implied the deficient suppression of Tfh functions and a consequent failure to repress the GC reactions, as observed in the HT samples. Moreover, using FACS and immunohistochemistry, Yamashita and colleagues showed increased the number of Tfh cells in HT tonsillar samples compared with RT samples^28^. Taken together, these findings also support the notion that B10 are indeed a committed functional lineage, as a blockage in the differentiation of these cells affects the proportion of the entire subset.

**Table 1:** Data of tonsils’ donors.

	Hypertrophied Tonsils (HT)	Recurrent tonsillitis (RT)	Hypertrophied Tonsils and Recurrent tonsillitis
*Number of patients*	5	5	2
*Gender*			
*Female*	3	4	1
*Male*	2	1	1
*Age in years (mean {range})*	4.2 {2–6}	13.4 {3–30}	Female: 16 Male: 6
*Other observations*			Male also suffered asthma

As it is long established that regulatory T cells (Tregs) are essential to tolerance^29, 30^, there have been studies characterizing tonsillar Tregs^31, 32^. However, tonsils are essentially B cell organs, which turn them in an excellent and novel model to study inflammatory and regulatory immune mechanisms from a human B cell perspective. In conclusion, we characterized IL10 secretion by tonsillar B cells, and the results contributed to a comprehensive picture of the cytokine-secreting B cell population in tonsils. The results suggest a role for the B10 population as a distinct lineage in the pathophysiology of HT. We provided evidence that defective B10 compartment indicates an increase in the proportion of GC, suggesting unrestrained Tfh function. Further mechanistic studies will define the accurate role of the B10 population. This study advances our understanding of the immune functions of the tonsils, a subject that has gained momentum not only in light of interests in the nasal route for vaccine administration to stimulate both mucosal and systemic immunity but also considering the prevalence of tonsillar diseases in childhood.

## Methods

### Isolation of cells

Primary human mononuclear cells were isolated from tonsils obtained from patients undergoing tonsillectomy. The total number of independent samples used for the different experiments accounted for N = 30. In the legend of each figure, we specified the number of patient samples used per experiment. Tonsillar mononuclear cells (TMC) were prepared in the following manner. Briefly, tonsils were collected in phosphate-buffered saline (PBS) containing 50 μg ml^−1^ amphotericin B (Richet, Buenos Aires, Argentina). The tissues were chopped using a scalpel in complete medium and passed through a 70-μm pore cell strainer (Falcon, San Jose, CA, USA). TMC were purified using density gradient centrifugation with Ficoll-Hypaque (GE Healthcare, Little Chalfont, UK). The viability of primary cells, determined using trypan blue exclusion, was greater than 99% in all preparations. Informed consent was obtained from subjects prior to the study. The institutional ethics committee (Clinical Hospital, School of Medicine, Buenos Aires and Institute of Otolaringology Arauz, Buenos Aires) approved the collection and use of clinical materials, conformed to the provisions of the Declaration of Helsinki (as revised in Edinburgh 2000). Therefore, all methods were performed in accordance with the relevant guidelines and regulations. Informed consent was obtained from all participants and/or their legal guardian/s. All experiments were performed with freshly isolated cells.

### Cell culture

Primary human B cells were cultured in IMDM medium (Life Technologies, Carlsbad, CA, USA) containing 10% heat-inactivated foetal calf serum, 2mM L-glutamine, 100 U/ml penicillin, 100 μg/ml streptomycin, 20 mM 4-(2-hydroxyethyl)-1-piperazineethanesulphonic acid buffer (HEPES), 1 mM sodium pyruvate and 50 μM 2-mercaptoethanol (all from Invitrogen, Carlsbad, CA, USA). Human IL2 (20 ng/ml; R&D Systems, Minneapolis, MN, USA) and human IL4 (20 ng/ml; R&D) were immediately added as supplements prior to initiating the experiments. Where indicated, human recombinant hCD40L (250 ng/ml; R&D Systems) and 25 μM CpG-ODN 2006 (InvivoGen, San Diego, CA, USA) were used. The cells were cultured at 1 × 10^6^ cells/ml in either 24-well (1 ml) or 48-well culture plates (0.5 ml).

### Antibodies and flow cytometry

Fluorochrome-conjugated mAbs specific for human CD1d (clone CD1d42), CD3 (clone SK7), CD20(clone L27), IgD(clone IA6-2), CD38 (clone HIT2), CD24 (clone ML5), CD39 (clone TU66), PDL-1 (clone MIH1), CD27 (clones M-T271 and Miltenyi clone LG.3A10), CD25 (clone M-A251), CD21 (clone B-ly4), CD5 (clone UCHT2), CD73 (clone AD2), CD62L (clone DREG-56), IL10 (clone JES3-9D7), IL6 (clone 8C9), IL8 (clone IT7F8), and IL17 (clone SCPL1382) and isotype control mAbs were purchased from BD Biosciences (San Jose, CA, USA), Biolegend (San Diego, CA, USA), Miltenyi (San Diego, CA, USA) and Immunotools (Friesoythe, Germany). For surface staining, TMC were incubated with excess mAb in PBS/1% BSA on ice for 40 min and washed in the same buffer. To evaluate the cytokines using intracellular staining, surface-stained cells were incubated with Cytofix/Cytoperm (BD PharMingen, San Jose, CA, USA) for 20 min in the dark and washed with Perm/Wash solution (BD PharMingen). Following permeabilization, the cells were stained with the respective anti-cytokine mAb. The cells were acquired using FACS Aria II (BD Biosciences) and analysed using FlowJo software (Treestar, Ashland, OR, USA). Single-stained controls were used to set compensation parameters. Fluorescence minus one and isotype-matched Ab controls were used to set analysis gates. Apoptotic and viable cells were distinguished based on differences in forward and side scatter, all results correspond to events within singlets and viable gates.

### Statistical analyses

The results were analysed using GraphPad Prism 5.0 software. The statistical analysis of the results was performed using the unpaired t test, and a p value of <0.05 was considered significant unless otherwise indicated.

## Electronic supplementary material

Supplementary information
