# Supplementary material for: Characterization of tonsillar IL10 secreting B cells and their role in the pathophysiology of tonsillar hypertrophy
Source: Sci Rep. 2017 Sep 11;7:11077. doi: 10.1038/s41598-017-09689-x (PMC5593840; doi:10.1038/s41598-017-09689-x)
Supplement: Supplementary file 1 — Supplementary information [file 41598_2017_9689_MOESM1_ESM.pdf]

Characterization of tonsillar IL10 secreting B cells and their role in the pathophysiology of tonsillar hypertrophy

Lindybeth Sarmiento Varon[1], Javier De Rosa[1], Andrés Machicote[1], Luis Ariel Billordo[1], Plácida Baz[1], Pablo Mariano Fernández[1,2], Isabel Kaimen Maciel[3], Andrés Blanco[4], and Eloísa Arana[1,2*]

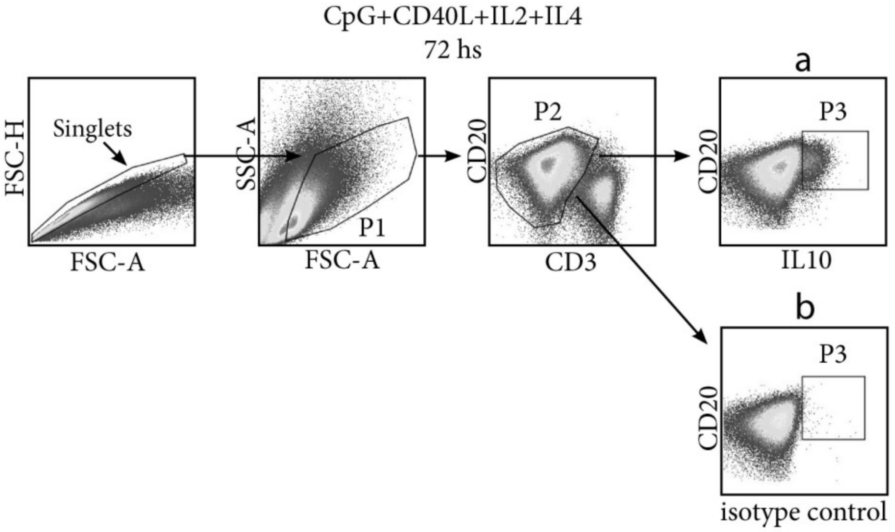

Supplemetary Figure 1. Detailed analysis performed to score the percentages of B10 shown in Fig 4a. TMC were cultured for 72 hs on CpG+CD40L+IL2+IL4 and PMA/ionomycin/Brefeldin A for the last 5 hs. The cells were subsequently stained for surface CD20 and CD3 plus either intracellular IL10 (a) or corresponding isotype control (b). P1 indicates living cells determined using FSC-SSC dot plot profiles within the singlets gate. P2 denotes $CD20^+$ cells. P3 denotes $CD20^+IL10^+$ cells.
